# Upregulation of S100A8 in peripheral blood mononuclear cells from patients with depression treated with SSRIs: a pilot study

**DOI:** 10.1186/s12953-023-00224-7

**Published:** 2023-12-04

**Authors:** Concepción Gamboa-Sánchez, Enrique Becerril-Villanueva, Samantha Alvarez-Herrera, Gabriela Leyva-Mascareño, Sandra L. González-López, Enrique Estudillo, Alberto E. Fernández-Molina, José Miguel Elizalde-Contreras, Eliel Ruiz-May, Aldo Segura-Cabrera, Janeth Jiménez-Genchi, Lenin Pavón, Sergio Roberto Zamudio, Gilberto Pérez-Sánchez

**Affiliations:** 1 Laboratorio de Psicoinmunología, Instituto Nacional de Psiquiatría Ramón de La Fuente Muñiz, Colonia San Lorenzo Huipulco, Calzada México-Xochimilco 101, Tlalpan, 14370 Ciudad de Mexico México; 2 Departamento de Fisiología, Escuela Nacional de Ciencias Biológicas, Instituto Politécnico Nacional. Unidad Profesional Adolfo López Mateos, Av. Wilfrido Massieu 399, Nueva Industrial Vallejo, Gustavo A. Madero, 07738 Ciudad de México México; 3 Laboratorio de Reprogramación Celular, Instituto Nacional de Neurología y Neurocirugía Manuel Velasco Suárez, Av. Insurgentes Sur 3877 Del. Tlalpan, 14269. Col. La Fama., Ciudad de México, México; 4 Red de Estudios Moleculares Avanzados, Instituto de Ecología A. C, Cluster BioMimic®, Carretera Antigua a Coatepec 351, Congregación El Haya, 91073 Xalapa, Veracruz México; 5 Present Address: Genomic Sciences, GSK, Stevenage, UK; 6 Hospital Psiquiátrico Fray Bernardino Álvarez. Av, Niño Jesús, San Buenaventura 214000, Tlalpan, Ciudad de Mexico México

**Keywords:** Depression, Biomarkers, S100A8

## Abstract

**Background:**

Major depressive disorder (MDD) affects more than 350 million people worldwide, and there is currently no laboratory test to diagnose it. This pilot study aimed to identify potential biomarkers in peripheral blood mononuclear cells (PBMCs) from MDD patients.

**Methods:**

We used tandem mass tagging coupled to synchronous precursor selection (mass spectrometry) to obtain the differential proteomic profile from a pool of PBMCs from MDD patients and healthy subjects, and quantitative PCR to assess gene expression of differentially expressed proteins (DEPs) of our interest.

**Results:**

We identified 247 proteins, of which 133 had a fold change ≥ 2.0 compared to healthy volunteers. Using pathway enrichment analysis, we found that some processes, such as platelet degranulation, coagulation, and the inflammatory response, are perturbed in MDD patients. The gene-disease association analysis showed that molecular alterations in PBMCs from MDD patients are associated with cerebral ischemia, vascular disease, thrombosis, acute coronary syndrome, and myocardial ischemia, in addition to other conditions such as inflammation and diabetic retinopathy.

**Conclusions:**

We confirmed by qRT-PCR that S100A8 is upregulated in PBMCs from MDD patients and thus could be an emerging biomarker of this disorder. This report lays the groundwork for future studies in a broader and more diverse population and contributes to a deeper characterization of MDD.

**Supplementary Information:**

The online version contains supplementary material available at 10.1186/s12953-023-00224-7.

## Introduction

Major depressive disorder (MDD) is a psychiatric illness characterized by mood disorders such as irritability, anhedonia, and sleep disturbance. It is accompanied by other cognitive, behavioral, and neurovegetative symptoms that significantly affect the individual's capability to have a normal life. Its high prevalence, recurrence, and enormous personal and social costs have been exacerbated due to the COVID-19 pandemic, making depression the leading cause of disability worldwide [1, 2]. As MDD is a multifactorial disease, a comprehensive approach is required for diagnosis and treatment. Currently, the diagnosis of MDD consists of an examination of the medical history, a physical examination, and the application of clinimetric tests [3]. However, several authors agree that clinimetric tests used for clinical follow-up of MDD patients are insufficient to establish a correlation between the clinimetric score and the pathophysiological condition of the patient. Given the complex etiology of MDD, the use of proteomic approaches can be helpful in the search for molecular alterations with the potential to be used as biomarkers for MDD. Proteomic analyses with mononuclear cells, plasma, or serum obtained from peripheral blood samples have increasingly impacted clinical research [4–7]. Notably, peripheral blood mononuclear cells (PBMCs) have great diagnostic power because of the high correlation observed at the transcriptomic level between these cells and the brain [8]. The identification of potential biomarkers of depression in PBMCs may help to improve the diagnosis of depression, stratify patients and personalize treatment. Therefore, the present report aimed to identify differentially expressed proteins (DEPs) in PBMCs from MDD patients using tandem mass labeling coupled to synchronous precursor selection (mass spectrometry). Pathway enrichment analysis and validation by qRT-PCR allowed us to enrich and strengthen the information obtained by mass spectrometry and to expand the knowledge about MDD.

## Materials and methods

### Subjects

The recruitment of patients with MDD and healthy volunteers was carried out at the Instituto Nacional de Psiquiatría Ramón de la Fuente Muñíz and the Hospital Psiquiátrico Fray Bernardino Álvarez (Mexico). This study was conducted according to the guidelines of the Declaration of Helsinki and approved by the Institutional Ethics and Research Committee of the Instituto Nacional de Psiquiatría Ramón de la Fuente Muñíz (Protocol INPRF-2035). The inclusion criteria consisted of patients diagnosed with severe MDD according to the DSM-IV-TR using the Spanish version of the 21-item Hamilton Depression Rating Scale (HDRS). All the participants received a detailed explanation of the study objectives and then signed the written declaration of consent. The patients were classified into two groups: patients with MDD without antidepressant treatment or having taken no antidepressants at least two months prior to the study (MDD group) and patients with MDD who were treated with antidepressants for at least one month (MDD + SSRI group). Only patients who took selective serotonin reuptake inhibitors (SSRIs) as medication were considered for this study; only two patients received benzodiazepines (BZ) in addition to SSRIs, as shown in Table 2. Their depressive episodes lasted no more than two years and had a minimum baseline score on the HDRS scale equal to or greater than 18. Eight healthy volunteers (HV group) between the ages of 18 and 60 years participated in this study and were considered the reference group.

### PBMC isolation

Peripheral blood samples (6 mL) were collected from MDD patients, MDD + SSRI patients, and healthy volunteers by venipuncture using sodium heparin vacuum tubes (Becton Dickinson Vacutainer®, USA). Then, PBMCs were isolated using Ficoll Histopaque (Sigma‒Aldrich, 10,771-500ML, USA) according to the manufacturer’s procedure. After obtaining the PBMCs, 1 mL TRIzol® Reagent (Invitrogen Life Technologies, 15,596–018, 200 ml) was added to each sample, and they were stored at –80 °C until protein and RNA extraction.

### Extraction of protein from PBMCs for proteomic analysis

Protein extractions were performed from PBMC samples using TRIzol® Reagent (Invitrogen Life Technologies, 15,596–018, 200 mL) according to the manufacturer’s instructions. The protein pellet was resuspended in 30 µL of 1% SDS and quantified using a Pierce BCA protein assay kit (Thermo Scientific PL212239).

### Extraction of RNA from PBMCs for qRT‒PCR analysis

RNA extractions were performed from PBMC samples using TRIzol® Reagent (Invitrogen Life Technologies) according to the manufacturer’s instructions. The RNA pellet was resuspended in 25 µL of ultrapure water, of which 2 µL was quantified using a NanoDrop spectrophotometer and stored at –80 °C until further use.

### PBMC protein pool formation

A protein pool of 100 μg was created for each study group (MDD, MDD + SSRIs, and HV). All participants were considered for the pool formation of their corresponding group. The pools were cleaned four times with 20% ice-cold trichloroacetic acid in acetone with 1 h of incubation at –20 °C between washes, followed by centrifugation at 13,000 × *g* for 15 min. The pellets were dried at room temperature for 5 min, resuspended in 8 M urea, and stored at –80 °C until mass spectrometry analysis.

### Trypsin digestion

Protein pool pellets were reduced with 10 mM Tris (2-carboxyethyl) phosphine (TCEP) at 60 °C for 45 min and alkylated with 30 mM iodoacetamide (IA) at room temperature in the dark for 1 h. Then, samples were quenched with 30 mM DTT for 10 min. Proteins were precipitated with acetone at –20 °C overnight. The mixture was centrifuged at 10,000 × *g* at 4 °C for 15 min. The supernatant was discarded, and the pellet was dried at room temperature for 15 min. The protein pellet was dissolved in 100 μL digestion buffer containing 50 mM triethylammonium bicarbonate and 0.1% SDS. Then, proteins were digested with trypsin (Trypsin Gold, Mass Spectrometry Grade, Promega, Madison, WI) at 37 °C overnight, followed by an additional trypsin digestion at 37 °C for 4 h.

### *Tandem mass tagging (TMT) of pools: MDD, MDD* + *SSRIs, and HV*

Peptides were labeled with TMT 6-plex reagents according to the manufacturer’s instructions (Thermo Fisher Scientific, Rockford, IL). We used the 126, 127 N, and 128C isobaric labels for MDD, MDD + SSRIs, and HV, respectively. Labeled samples were pooled and fractionated using strong cation exchange cartridges (SCX) (Thermo Scientific, Bellefonte, PA, USA). Four fractions were collected based on the different concentrations of elution buffer, including 150, 250, and 500 mM KCl. Each fraction was desalted with C18 cartridges and dried using a CentriVap (Labconco Kansas, Missouri).

### *Tandem mass tagging (TMT) coupled to synchronous precursor selection (SPS)-MS*^*3*^*(TMT-SPS-MS*.^*3*^*)*

Nano LC‒MS/MS analysis and synchronous precursor selection (SPS)-MS3 were performed. We used an Orbitrap Fusion Tribrid mass spectrometer (Thermo-Fisher Scientific, San Jose, CA) equipped with an EASY-Spray nano ion source (Thermo-Fisher Scientific, San Jose, CA). The mass spectrometer was connected to an UltiMate 3000 RSLC system (Dionex, Sunnyvale, CA). Each sample was reconstituted with 0.1% formic acid in LC‒MS grade water (solvent A), and 5 μL was injected into a nanoViper C18 trap column (3 µm, 75 µm × 2 cm, Dionex) at a 3 μL/min flow rate and then separated on an EASY-Spray C-18 RSLC column (2 µm, 75 µm × 25 cm) using a 100-min gradient with a flow rate of 300 nL/min and solvent A and solvent B (0.1% formic acid in 90% acetonitrile). The gradient was as follows: solvent A for 10 min, 7–20% solvent B within 25 min, 20% solvent B for 15 min, 20–25% solvent B for 15 min, 25–95% solvent B for 20 min, and solvent A for 8 min. The mass spectrometer was operated in positive ion mode with a nanospray voltage of 3.5 kV and a source temperature of 280 °C. External calibrators included caffeine, Met-Arg-Phe-Ala (MRFA) and Ultramark 1621. Full MS scans in the Orbitrap analyzer were at 120,000 (FWHM) resolution, scan range of 350–1500 m/z, AGC of 2.0e5, maximum injection time of 50 ms, intensity threshold of 5.0e3, dynamic exclusion 1 at 70 s, and 10 ppm mass tolerance. For the MS2 analysis, the 20 most abundant MS1 were isolated with charge states set to 2–7. The fragmentation parameters included CID with 35% collision energy and activation Q of 0.25, AGC of 1.0e4 in maximum injection time of 50 ms, precursor selection mass range of 400–1200 m/z, precursor ion exclusion with low 18 m/z and high 5 m/z, and isobaric tag loss TMT; the detection was performed in the ion trap. Afterwards, MS3 spectra were acquired as previously described [9] using SPS of 10 isolation notches. The MS3 precursors were fragmented by HCD with 65% collision energy and analyzed using the Orbitrap with a resolution of 60,000 at 120,500– m/z scan range, 2 m/z isolation window, 1.0e5 AGC, and maximum injection time of 120 ms with 1 microscan.

### *Bioinformatic analysis from TMT-SPS-MS*^*3*^* data*

The raw data were processed using Proteome Discoverer 2.1 (PD, Thermo Fisher Scientific Inc.). The following searches were carried out with the Mascot server (version 2.4.1, Matrix Science, Boston, MA), AMANDA [9] and SQUEST HT [10]. The search with both engines was conducted against the UniProt *Homo sapiens* reference proteome database [10, 11]. Parameters in the search included full tryptic protease specificity and two missed cleavages allowed. In addition, static modifications included the carbamidomethylation of cysteine (+ 57.021 Da) and TMT 6-plex N-terminal/lysine residues (+ 229.163 Da). Furthermore, dynamic modifications included methionine oxidation (+ 15.995 Da) and deamidation in asparagine/glutamine (+ 0.984 Da). Tolerances of ± 10 ppm and ± 0.6 Da were used for the SPS-MS3 method, in which the identification was performed with lower resolution in the linear ion trap. The resulting peptide hits were filtered for a maximum 1% FDR using the Percolator algorithm [12]. The TMT 6-plex quantification method within PD software was used to calculate the reporter ratios at a mass tolerance of ± 10 ppm with the most confident centroid, and a precursor coisolation filter of 45% was applied. In the SPS-MS3 method, quantification was performed at the MS3 level.

### Pathway enrichment analysis

Differentially expressed proteins across different groups of donors were used to perform a pathway enrichment analysis with the ClusterProfiler R package [13]. The analysis was performed on gene sets from biological processes of the Gene Ontology (GO) database [14]. The pathway enrichment analysis for gene-disease associations from differentially expressed proteins was performed using the DisGeNET database [15].

### S100A8 and S100A9 qRT‒PCR

S100A8 and S100A9 were tested by quantitative RT‒PCR (qRT‒PCR) in individual samples from the three groups (MDD, MDD + SSRIs, and HV). cDNA was synthetized from 1 µg of total RNA (from PBMCs) (pretreated with DNAse; Invitrogen) using 1 µL (200 U) of MMLV reverse transcriptase (Invitrogen). Quantitative PCR was performed using 50 ng of cDNA as a template. The probes used were 219,726,190 (S100A8) and 219,726,194 (S100A9) from IDT technologies and Hs01060665_g1 (β-actin) and TaqMan® Master Mix, both from ThermoFisher®. All assays were performed in duplicate using CFX96TM Real Team System® (Bio-Rad). The PCRs were performed at a final volume of 3.5 µL. The CFX96™ was set at 95 °C for 10 min (step 1), followed by 95 °C for 15 s and 60 °C for 60 s (step 2). Step 2 was programmed for 40 cycles. The quantitative analysis was performed with the 2^−ΔΔCt^ method [16] using the ΔCt mean of healthy volunteers as a calibrator to calculate the ΔΔCt.

### Statistical analysis

Statistical analyses were performed using GraphPad Prism 9.1.1. The results are shown as the mean with SEM (qRT-PCR) or SD (Demographic data), and Kolmogorov‒Smirnov was used as a normality test. For the comparison between groups, we used the Kruskal‒Wallis and Dunn’s multiple comparisons tests. The results were considered significant at *p* < 0.05.

## Results

### Participant recruitment and demographic data

Eight healthy volunteers (HV), nine MDD patients without treatment (MDD), and twelve MDD patients with SSRI treatment (MDD + SSRI) fulfilled the inclusion criteria for the proteomics analysis. The demographic characteristics of the participants are depicted in Table 1, while the treatment details are described in Table 2.

**Table 1:** Demographic data and psychiatric tests

	HEALTHY VOLUNTEERS (HV)	MDD PATIENTS	MDD + SSRIs PATIENTS	
	MEAN(SD)	MEAN(SD)	MEAN (SD)	p
FEMALE	2	4	12	
MALE	6	5	0	
TOTAL	8	9	12	
AGE (YEARS)	29.63(5.181)	34.67(9.695)	39.08(11.45)	*p* > 0.05
BMI	27.16(2.916)	23.19(2.862)*	24.89(2.382)	*; *p* < 0.05 *vs* HV
HDRS	0.875(1.642)	28.44(6.766)*	28.75(5.987)*	*; *p *< 0.05 *vs* HV

**Table 2:** Antidepressant treatment of MDD + SSRIs group

TIME OF TREATMENT	TYPE OF TREATMENT	DOSE	AGE	BMI	HDRS
3 months	SSRIs	50 mg	38	21.504	38
5 months	SSRIs	20 mg	52	29.727	29
46 months	SSRI	20 mg	54	27.392	31
3 months	SSRIs/BZ	50 mg/1 mg	29	24.201	40
2 months	SSRIs	50 mg	22	26.093	22
2 months	SSRIs	100 mg	22	25.778	28
1 months	SSRIs/BZ	80 mg/ 2.5 mg	31	27.026	25
2 months	SSRIs	50 mg	47	24.456	31
48 months	SSRIs	40 mg	45	23.424	31
5 months	SSRIs	10 mg	36	22.318	24
1 months	SSRIs	50 mg	53	23.968	26
2 months	SSRIs	50 mg	40	22.833	20

### *Differential proteomic profile by TMT-SPS-MS*.^*3*^

The proteomic analysis by TMT-SPS-MS^3^ led us to identify 247 proteins in MDD patients (-/ + SSRIs) compared to healthy volunteers (Supplementary Table 1), of which 133 were found to be differentially expressed (DEPs) with a fold change ≥ 2.0 (MDD/HV). Some of these DEPs are reported for the first time in PBMCs from patients with MDD in this work and with an abundance ratio ≥ 2 (Table 3).

**Table 3:** DEPs reported for the first time in PBMC from patients with MDD and with an abundance ratio ≥ 2

Gene Name	Protein Accession number	Abundance ratio: MDD/HV	Mascot score	Amanda score
AHSG	C9JV77	6.83	96.37	948.42
CA1	P00915	5.74	49.25	433.17
AGP2 (ORM2)	P19652	5.58	230.23	1933.58
YWHAZ	P63104	4.46	402.74	3032.29
AGP1 (ORM1)	P02763	4.29	397.76	3448.21
C4BPA	P04003	4.25	108.15	784.74
LSP1	P33241	3.94	62.32	838.32
SNCA	P37840	3.87	64.53	501.95
S100A8	P05109	3.87	130.05	1022.72
S100A9	P06702	2.25	71.97	870.94
CAVIN2 (SDPR)	O95810	3.60	158.38	2049.49
APOA1	P02647	3.50	146.60	1001.43
SELP	P16109	3.39	107.16	883.26
PGK1	P00558	2.10	545.62	2903.95
C3	P01024	2.30	553.35	5177.64

### Pathway enrichment analysis

#### Biological processes

**Fig. 1:**
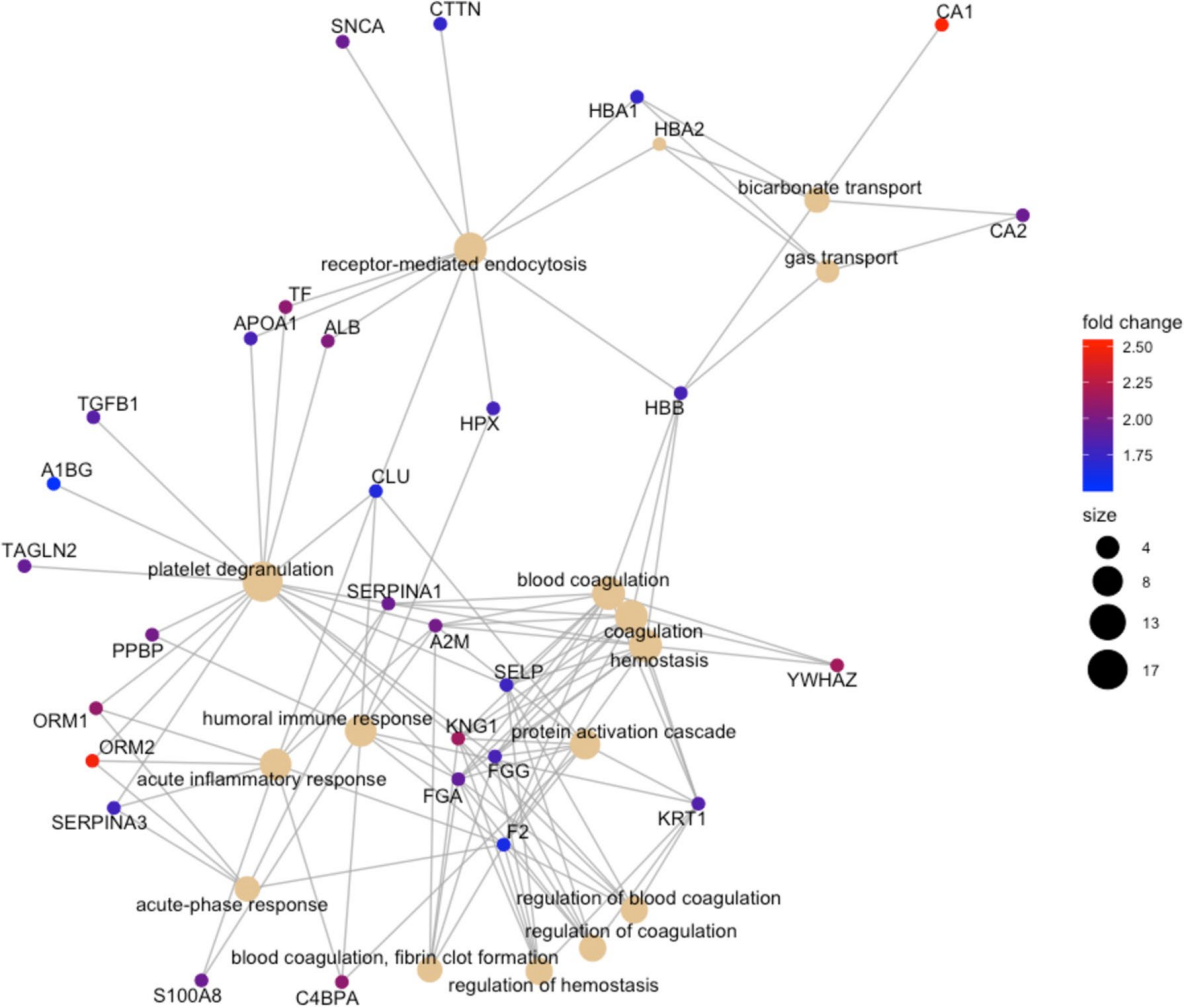
The most significant biological processes that are perturbed in MDD vs HV. The color palette shows the log_2_ fold change for each GO annotation (protein), while the size of black nodes (right panel) is a visual scale that represent the number of GO annotations for each GO term (biological process)

**Fig. 2:**
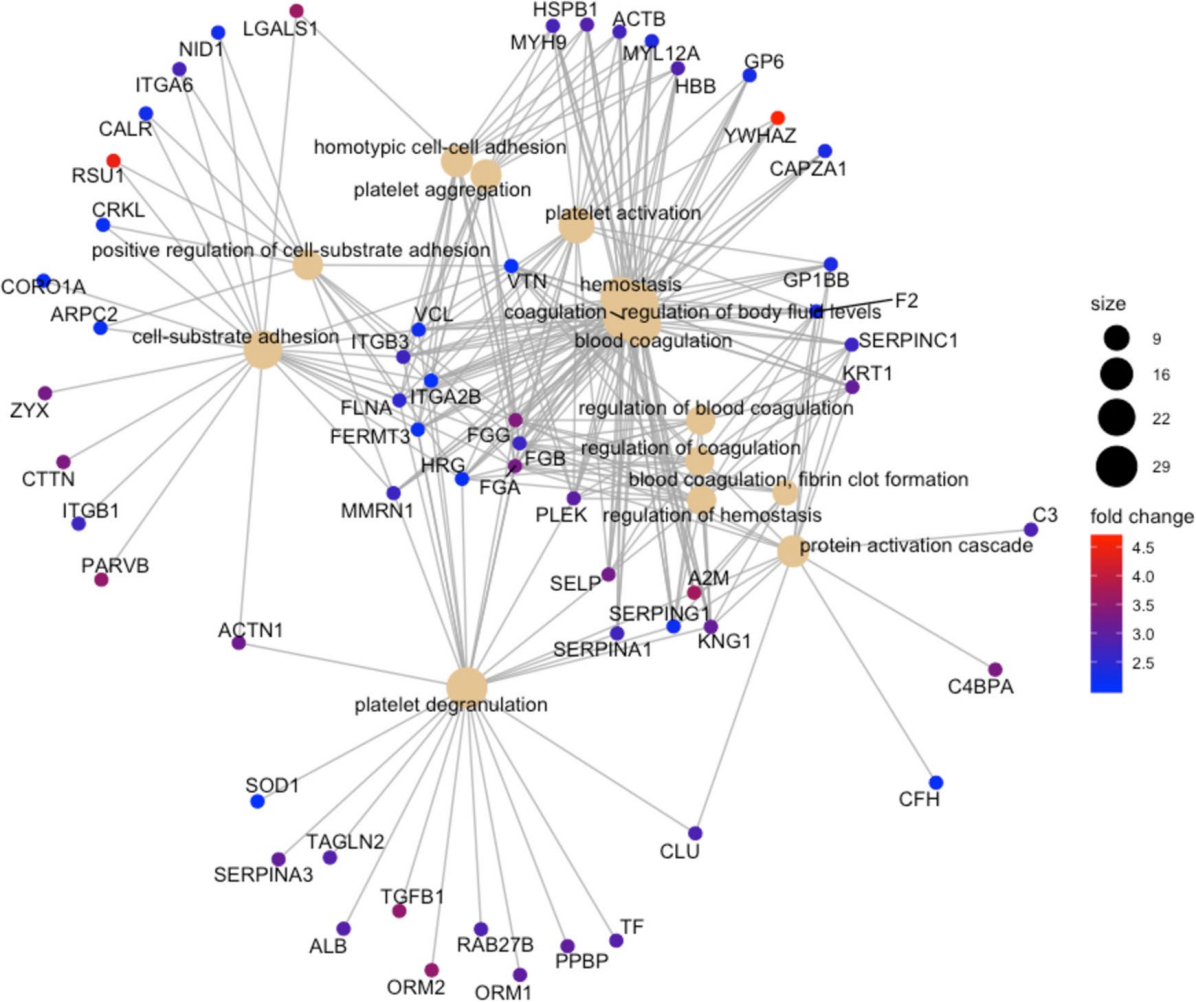
The most significant biological processes that are perturbed in MDD + SSRI vs HV. The color palette shows the log_2_ fold change for each GO annotation (protein), while the size of black nodes (right panel) is a visual scale that represent the number of GO annotations for each GO term (biological process)

**Fig. 3:**
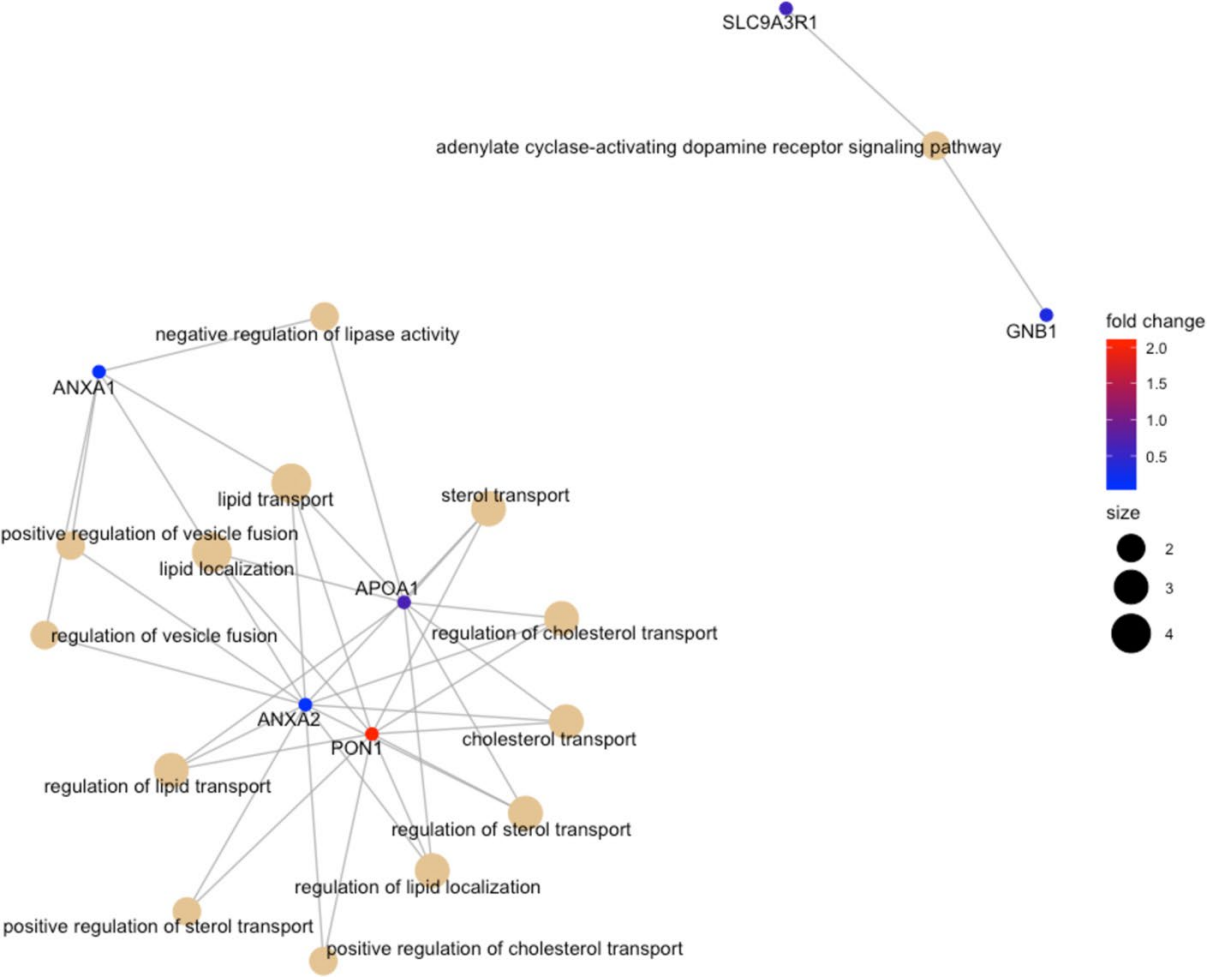
The most significant biological processes that are perturbed in MDD vs MDD + SSRIs. The color palette shows the log_2_ fold change for each GO annotation (protein); while the size of black nodes (right panel) is a visual scale that represent the number of GO annotations for each GO term (biological process)

The pathway enrichment analysis of the differentially expressed proteins obtained from the TMT-SPS-MS^3^ analysis revealed the most significant biological processes that are perturbed in MDD (Figs. 1, 2 and 3).

#### Gene-disease associations

**Fig. 4:**
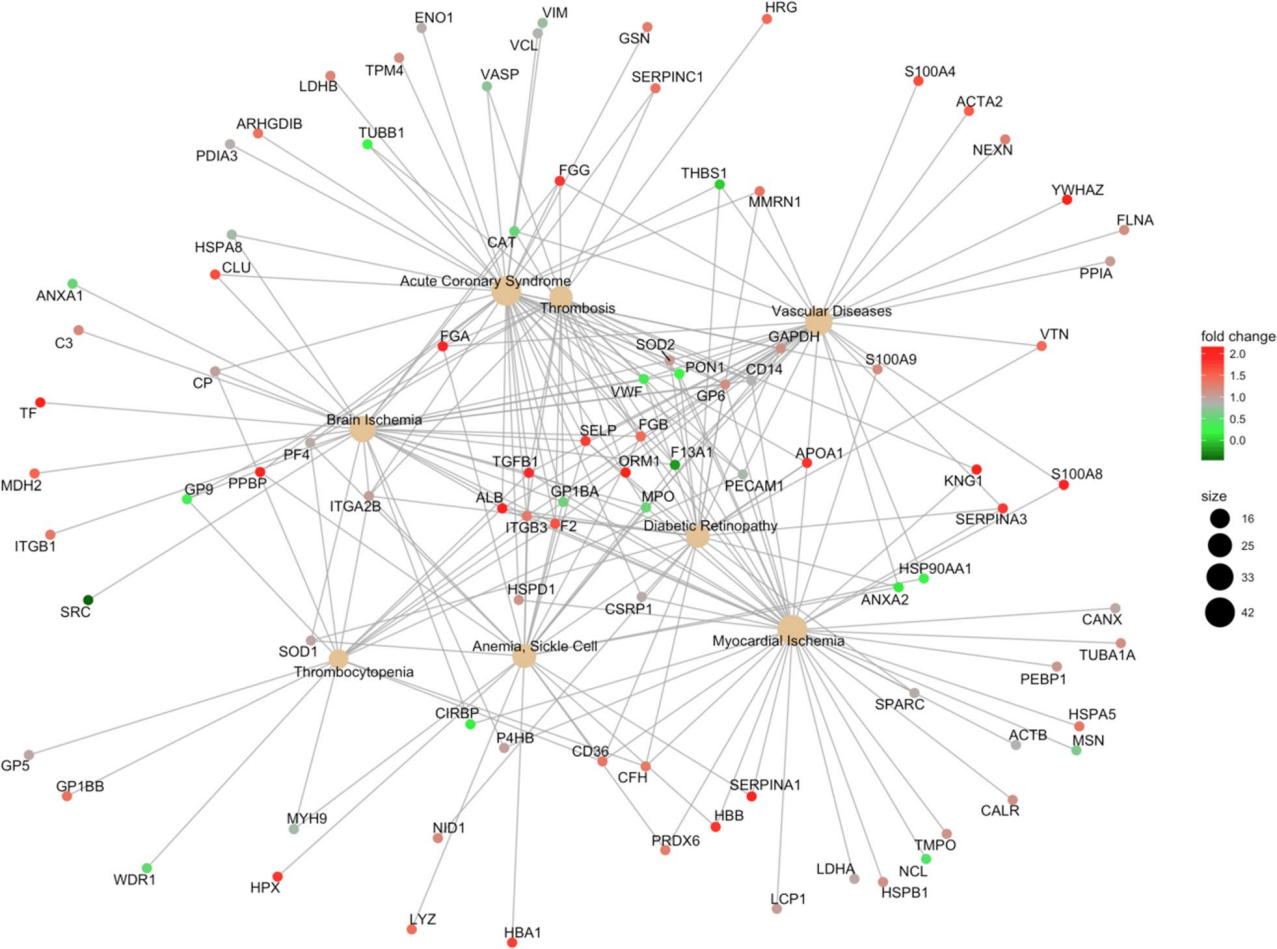
Gene-disease associations for MDD. The color palette shows the log_2_ fold change for each GO annotation (protein), while the size of black nodes (right panel) is a visual scale that represent the number of GO annotations for each GO term (disease)

Conditions such as brain ischemia, vascular diseases, thrombosis, acute coronary syndrome, myocardial ischemia, thrombosis, anemia, and diabetic retinopathy were associated with MDD (Fig. 4).

### Gene expression of S100A8 and S100A9

**Fig. 5:**
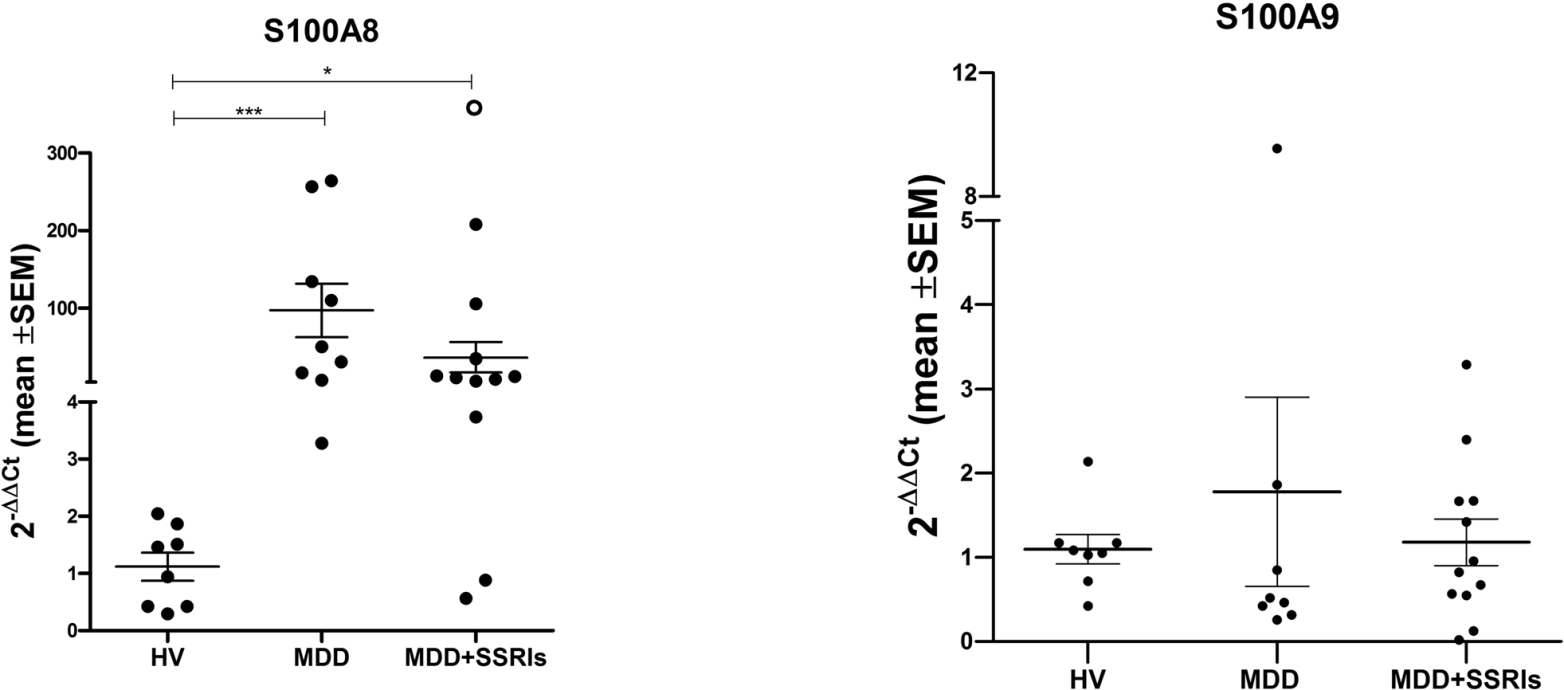
S100A8 and S100A9 qRT-PCR. A) mRNA levels of S100A8 in PBMC from HV, MDD patients and MDD + SSRIs patients; B) mRNA levels of S100A9 in PBMC from HV, MDD patients and MDD + SSRIs patients. Statistical analysis was performed by Kruskall- Wallis with Dunn’s post hoc (*, *p* < 0.05; ***, *p* < 0.001). The open circle is a datum that is outside the axis limit

The validation by qRT‒PCR showed significantly higher levels of S100A8 mRNA in MDD (*p* < 0.001) and MDD + SSRI patients (*p* < 0.05) than in healthy volunteers (Fig. 5A). We did not detect differences in the mRNA levels of S100A9 between groups (*p* = 0.4594) (Fig. 5B).

**Fig. 6:**
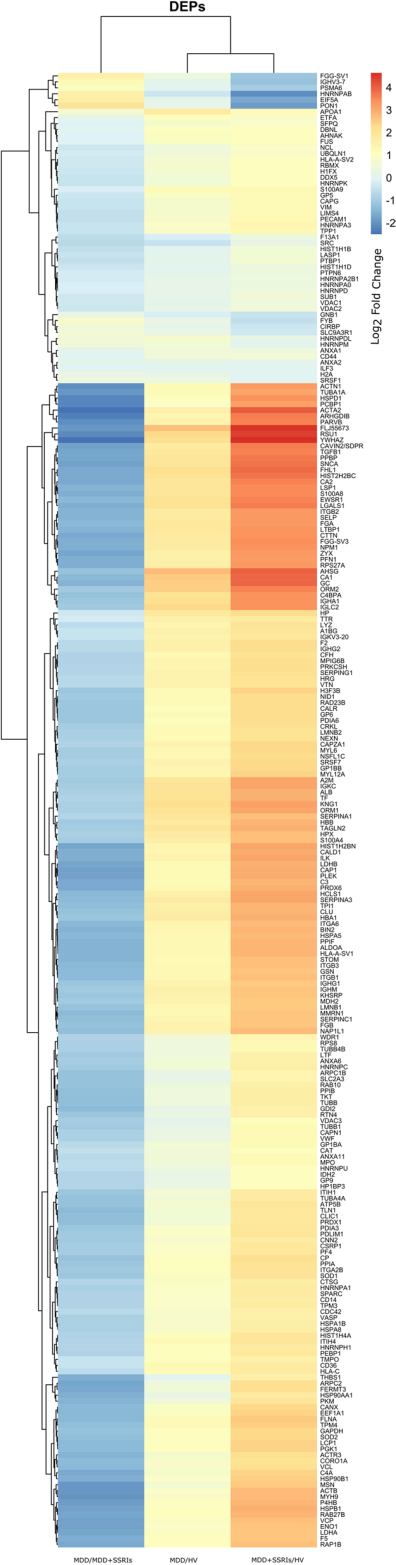
Heat map showing the differential expression pattern in the three study groups. The color palette shows the Log_2_ fold change for each DEP

In Fig. 6, we summarized all DEPs found in this work in a heat map, showing their differential expression pattern in the three study groups. Clustering was performed utilizing RStudio and the pheatmap v1.012 package with the euclidean distance and complete method.

## Discussion

In this work, we present a set of differentially expressed proteins that could be potential biomarkers of MDD, some of them being reported for the first time in PBMCs from MDD patients. Using pathway enrichment analysis, we revealed the most significant biological processes perturbed in MDD patients, as well as some conditions associated with MDD.

AGP1 and AGP2 belong to the plasma α-acid glycoprotein family, also known as orosomucoid proteins (ORM1 and ORM2). α-AGP proteins participate in the binding and transport of drugs and are upregulated in cancer and inflammatory diseases [17]. AGP1 is increased in plasma during the acute phase of depression, in which it positively correlates with high concentrations of IL-6 and C-reactive protein (CRP) [18]. Other works have suggested that AGP1 could be a predictor of response to antidepressants [19, 20]. In line with these studies, we report increases in these proteins in PBMC from MDD patients; and this is the first report that associates AGP2 with depression.

CA1 has shown increases in the cingulate cortex of MDD patients; similarly, CA1 and SNCA gene expression was found to be elevated in peripheral blood leukocytes of remitters with respect to nonremitters with geriatric depression [21, 22]. Similarly, PGK1, P-selectin (SELP), and APOA1 have also been reported to be elevated in the serum of MDD patients, which agrees with our results [23–30]. In contrast to our data, serum LSP1 protein decreases after antidepressant treatment [31]. YWHAZ, which encodes the 14–3-3 protein, has been found to be altered in patients with MDD, bipolar disorder, and schizophrenia [32]. In particular, YWHAZ has been shown to have a differential expression pattern in responder patients compared to nonresponders to antidepressants [33], and it has been proposed as a biomarker for antidepressant treatment response.

Consistent with previous reports, we also found alterations in some components of the complement system, such as C3 and C4BPA. In previous studies, plasmatic C3 and C3a was reported increased in MDD patients [34], whereas C5 and C4 were elevated in the cerebrospinal fluid [35] and serum [36], respectively; however, more studies are needed to elucidate its role in MDD.

Alpha 2-HS glycoprotein (AHSG) was the protein with the highest fold-change in patients with MDD (Table 3). It has been reported that AHSG protein levels are elevated in the prefrontal cortex and hippocampus of rats under chronic mild stress (CMS), as well as in the serum of IL-18-deficient mice with a depression-like phenotype [37], suggesting a potential role of AHSG in MDD. We also showed that CAVIN2 was increased in MDD patients. Both CAVIN2 (SDPR) and CAVIN 3 are regulators of the circadian rhythm, which is often disturbed in patients with MDD [38]. Furthermore, two single nucleotide polymorphisms in CAVIN 2 have previously been associated with depression [39].

It is known that S100 proteins are involved in several inflammatory processes and work as calcium chelators. S100A8 and S100A9, also known as MRP8 and MRP14, respectively, are increased in serum from mice under chronic unpredictable mild stress (CUMS) [40]. In contrast, S100A4 has been described as a neuroprotector during brain injury [41]. In this work, we observed high protein levels of S100A8, S100A9, and S100A4 in MDD and MDD + SSRIs patients. Additionally, S100A8 and S100A9 are considered biomarkers of poor prognosis in inflammatory and metabolic diseases as well as some types of cancer. During an infection or inflammatory disease, S100 proteins exacerbate the inflammatory response by increasing the production of proinflammatory cytokines in immune cells in an autocrine and paracrine manner through the interaction with TLR4 [42]. The overexpression of S100A8 and S100A9 in primary cultures induces the production of reactive oxygen species (ROS) and IL-10 mRNA [43]. Calprotectin, an S100A8/S100A9 dimer, induces the production of ROS, iNOS and proinflammatory cytokines in the brain, exacerbating apoptosis in oligodendrocyte precursor cells via the NFκB pathway [44]. Through the regulation of antioxidant enzymes, S100A8 promotes the production of ROS, which stimulates NFκB to generate proinflammatory cytokines, particularly IL-1β and TNF-α, as well as molecules involved in apoptosis [45]. In 2018, Gong et al. (2018) demonstrated that the intracranial administration of S100A8/A9 in the hypothalamus of mice with CUMS increased the production of proinflammatory cytokines at both the cerebral and peripheral levels [46, 47]. Stankiewicz et al. proposed the hypothesis that chronic stress in mice promotes an increase in blood pressure, which results in endothelial microdamage and overexpression of S100A8 and S100A9 [47]. Fluoxetine provokes an increase in the levels of intracellular calcium, which in turn stimulates S100A8 and S100A9 production [48, 49]. Hence, the levels of S100A8 and S100A9 have a positive correlation with those of intracellular calcium [50].

Since S100A8 and S100A9 are involved in inflammation, which is related to depression, we selected them for validation by qRT‒PCR on individual samples from each participant, as opposed to proteomic analysis performed from a pool. We confirmed that S100A8 was higher in MDD and MDD + SSRIs patients than in healthy volunteers but found no difference between MDD + SSRIs and MDD. It should be noted that the drugs administered, doses, and treatment times of the MDD + SSRIs group were very heterogeneous, and we need more studies in a larger cohort to clarify the effects of SSRIs on the expression of S100A8 in PBMCs of these patients. Importantly, to our knowledge, this is the first work to report upregulation of S100A8 in PBMCs from MDD patients. In contrast, S100A9 did not show changes in its gene expression, and this issue will be further explored in future work.

On the other hand, the pathway enrichment analysis revealed that MDD patients present molecular disturbances in processes associated with hemostasis, such as platelet processes and blood coagulation. These disturbances match the results obtained from the analysis of gene-disease associations, which revealed that MDD patients have underlying molecular alterations associated with conditions such as thrombosis, thrombocytopenia, cerebral ischemia, vascular diseases, acute coronary syndrome, and myocardial ischemia. The link between platelet and coagulation-related disorders and the pathophysiology of depression is widely supported in the literature. It is known that patients with MDD show an increase in their platelet activity and platelet aggregation [51], two critical events for hemostatic plug formation and thrombosis, which in turn increases the risk of conditions such as vascular diseases, acute coronary syndrome, and myocardial and brain ischemia.

An interesting fact is that patients with depression are more likely to develop cardiovascular disease and vice versa, and the presence of both in a patient worsens the prognosis and increases mortality [52]. Likewise, a high percentage of those who suffer from brain ischemia (ischemic stroke) or myocardial ischemia also suffer from depression [53, 54], and their rehabilitation is more complicated with a worse prognosis [55, 56]. The alterations in lipid metabolism observed in MDD *vs* MDD + SSRI patients suggest that this is an effect of SSRI medication. In fact, fluoxetine, a widely used SSRI drug, induces lipid metabolism changes [57]. Serum paraoxonase 1 (PON1), an enzyme with antioxidant potential that plays a role in lipid metabolism [58], was increased in MDD compared to MDD + SSRI patients. In addition, PON1 polymorphisms have been associated with the occurrence of depression [59, 60], although this is not entirely clear.

Other conditions, such as diabetic retinopathy, anemia, and inflammation processes, are also highlighted in MDD patients. Disturbances in their inflammatory profiles are well documented [61–63], and several works suggest that these alterations contribute to depression; however, the precise mechanism remains unclear.

Numerous studies have explored the search for biomarkers in serum and plasma samples. In recent years, we have prioritized the identification of new research targets, including PBMCs. PBMCs offer a new perspective on the interaction between the nervous, immune, and endocrine systems due to their close connection and communication. This information demonstrates how alterations in one system can impact other two, and such imbalances can be detected in cells from various systems, including the immune system [64, 65]. These changes have the potential to be investigated as potential biomarkers following a series of validations. While the monoamine hypothesis was once considered one of the first and most convincing explanations of the etiopathogenesis of depression, which solely focused on the nervous system, current studies present an integrative hypothesis [66]. According to this integrative approach, both biochemical factors (e.g., hormones, neurotransmitters, growth factors, pro- and anti-inflammatory cytokines) and environmental, genetic, and psychosocial factors may contribute and play a role in the pathophysiology of depression. Examining depression patients through immune system cells provides a novel and distinct perspective compared to the traditional serum or plasma approach; moreover, several authors have proposed PBMC as a useful tool for searching biomarkers of neuropsychiatric disorders [8, 67–69].

In conclusion, we identified a link between depression and several biological processes and diseases. This link may also explain the great complexity of the pathophysiology of depression and its high rates of comorbidity with several pathological conditions. Furthermore, we demonstrate that S100A8 is upregulated in PBMCs from patients with MDD and could be an emerging biomarker of this disorder. The future of this pilot study lies in expanding the sample size and testing more potential biomarkers from our proteomic database.

### Limitations of the study

The cohort was small in this initial approach. In future study protocols, we recommend prioritizing cohort size and validating each protein individually, as we did with S100A8 and S100A9.

In this study the biological replicates are found in the number of individuals in each pool, which allowed us to obtain an acceptable sample quantity for a single shot gun analysis. Despite the limitations, it is crucial to make the generated information available to the scientific community. The BMI was statistically lower in the MDD group than in HV; this happened when we separated the patients into two groups: MDD and MDD + SSRI.

### Supplementary Information

**Additional file 1: Supplementary Table 1.**

## Data Availability

All data presented in this study are available in the supplementary material and/or can be requested directly from the corresponding author.
